# Plasmonic color filter array based visible light spectroscopy

**DOI:** 10.1038/s41598-021-03092-3

**Published:** 2021-12-08

**Authors:** Jyotindra R. Shakya, Farzana H. Shashi, Alan X. Wang

**Affiliations:** School of Electrical Engineering and Computer Science, Oregon State University, 1148 Kelley Engineering Center, Corvallis, OR 97331 USA

**Keywords:** Applied optics, Optoelectronic devices and components, Optical spectroscopy

## Abstract

Compared with traditional Fabry–Perot optical filters, plasmonic color filters could greatly remedy the complexity and reduce the cost of manufacturing. In this paper we present end-to-end demonstration of visible light spectroscopy based on highly selective plasmonic color filter array based on resonant grating structure. The spectra of 6 assorted samples were measured using an array of 20 narrowband color filters and detected signals were used to reconstruct original spectra by using new unmixing algorithm and by solving least squares problem with smoothing regularization. The original spectra were reconstructed with less than 0.137 root mean squared error. This works shows promise towards fully integrating plasmonic color filter array in imagers used in hyperspectral cameras.

## Introduction

Visible Light Spectroscopy has many applications including cancer detection^1–3^, remote sensing^4–6^, mineralogy^7^, food safety^8^ and artwork authentication and preservation^9–12^. Conventional RGB camera can provide only 3 color information, which is often insufficient to detect sharp features in spectrum. Hence cameras with many color channels are often used for spectroscopy. Such imaging systems are called hyperspectral cameras which has many widespread usages. Visible light spectroscopy when used in imaging modality rather than spot spectroscopy allows computational algorithms to take advantage of spatial-spectral information such as spectral similarity in neighboring spatial pixels. Various algorithms have been proposed for reconstructing sample spectrum from hyperspectral images which take advantage of spatial-spectral information^13–21^. Hence improvements in hyperspectral imagers and cameras are essential towards progression in visible light spectroscopy. With development and advancement of technology, the hyperspectral cameras have been miniatured into form factors that are similar to regular cameras. The hyperspectral camera can use various types of color filters such as dye based filters, Fabry–Perot based filters and plasmonic color filters. The state-of-the-art hyperspectral CMOS camera^22,23^ uses Fabry–Perot based Bayer color filter array^24^. To fabricate many spectral filters, however, it takes as many processing and lithography steps as the number of spectral bands desired. In contrast, plasmonic color filters can be fabricated in single lithographic step regardless of the number of spectral bands. This is because the spectral peaks in plasmonic color filters depend only on lateral dimension. Integration of plasmonic color filters in CMOS imagers greatly reduce cost of the imagers and overall hyperspectral cameras. Due to these benefits, there has been many publications of plasmonic color filters including hole arrays^25–28^, patch arrays^29^ and 1-D gratings^30–32^. Although hole arrays and patch arrays can be used as color filters, due to the wide transmission peak, it is not suitable when high spectral resolution is needed. Furthermore, plasmonic color filters with resonant grating structure provides much narrower transmission spectra^33,34^ and hence allows for resolving spectral features even with sharp edges. There are many publications on plasmonic color filters using gold and silver in infrared region. Due to the high loss and inferior plasmonic properties, gold and silver do not perform well in the visible range, especially towards shorter wavelengths. Hence aluminum can be used as plasmonic material in visible range. In addition to the lower loss in visible range, low fabrication cost, mature processing and compatibility with CMOS process makes aluminum a good choice as plasmonic material for integration with CMOS imager. In this paper, an end-to-end demonstration of visible light spectroscopy using aluminum plasmonic color filter array is presented.

### Advantages of resonant grating structure

**Figure 1:**
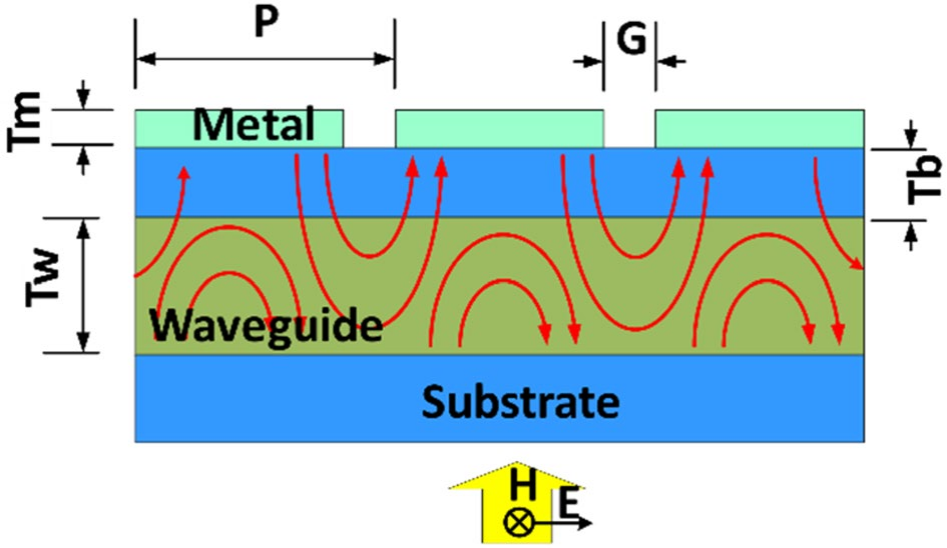
Schematic of Plasmonic Grating Filter with buried slab waveguide with illustrative E-field lines in red and the annotated parameters. Period of grating is P (260 nm to 450 nm at 10 nm steps), gap between fingers is G (60 nm), thickness of the metal is Tm (40 nm), thickness of the waveguide is Tw (90 nm) and distance between grating and waveguide is Tb (60 nm). The metal is Aluminum, the waveguide is made of Silicon Nitride layer buried in Silicon Dioxide and substrate is Silicon Dioxide.

Plasmonic color filters can be designed using either 1-D (grating) or 2-D structure (hole array or patch array). Due to periodic structure, these structures have counter propagating SPP modes due to Bragg reflection, which forms standing SPP modes in the periodic structure. However, such modes are highly lossy due to conductive losses in the metals used, which leads to broader resonant peaks and less spectral selectivity. An addition of slab waveguide under the grating provides means to periodically replenish energy into the resonant modes hence enhancing Q-factor. The standing SPP mode in the grating couples to the guided mode in the slab waveguide based on evanescent wave coupling. Such plasmonic grating filters with buried slab waveguide have narrower resonant peaks compared to conventional plasmonic grating filters. The distance to the slab waveguide can be tuned to optimize the design of such structure. If the slab waveguide is very close to metal structure, the guided mode inside the slab waveguide becomes lossy and hence the resonant peaks become broader. If the slab waveguide is too far, the coupling becomes less efficient, and the structure gradually changes towards conventional plasmonic grating filter without waveguide. Figure 1 shows the schematic of resonant grating structure and corresponding dimensions. The resonant grating structure has narrow passband in the range of 10–20 nm, which allows for high resolution spectroscopy. Due to complex nature of coupling between the grating and slab waveguide, there is no analytical method (to our knowledge), to design and optimize such structure without resorting to EM solvers. Hence the filter structure (Fig. 1) was designed and optimized using Rigorous Coupled Wave Analysis (RCWA) technique.

## Results

### Filter array design and simulation

**Figure 2:**
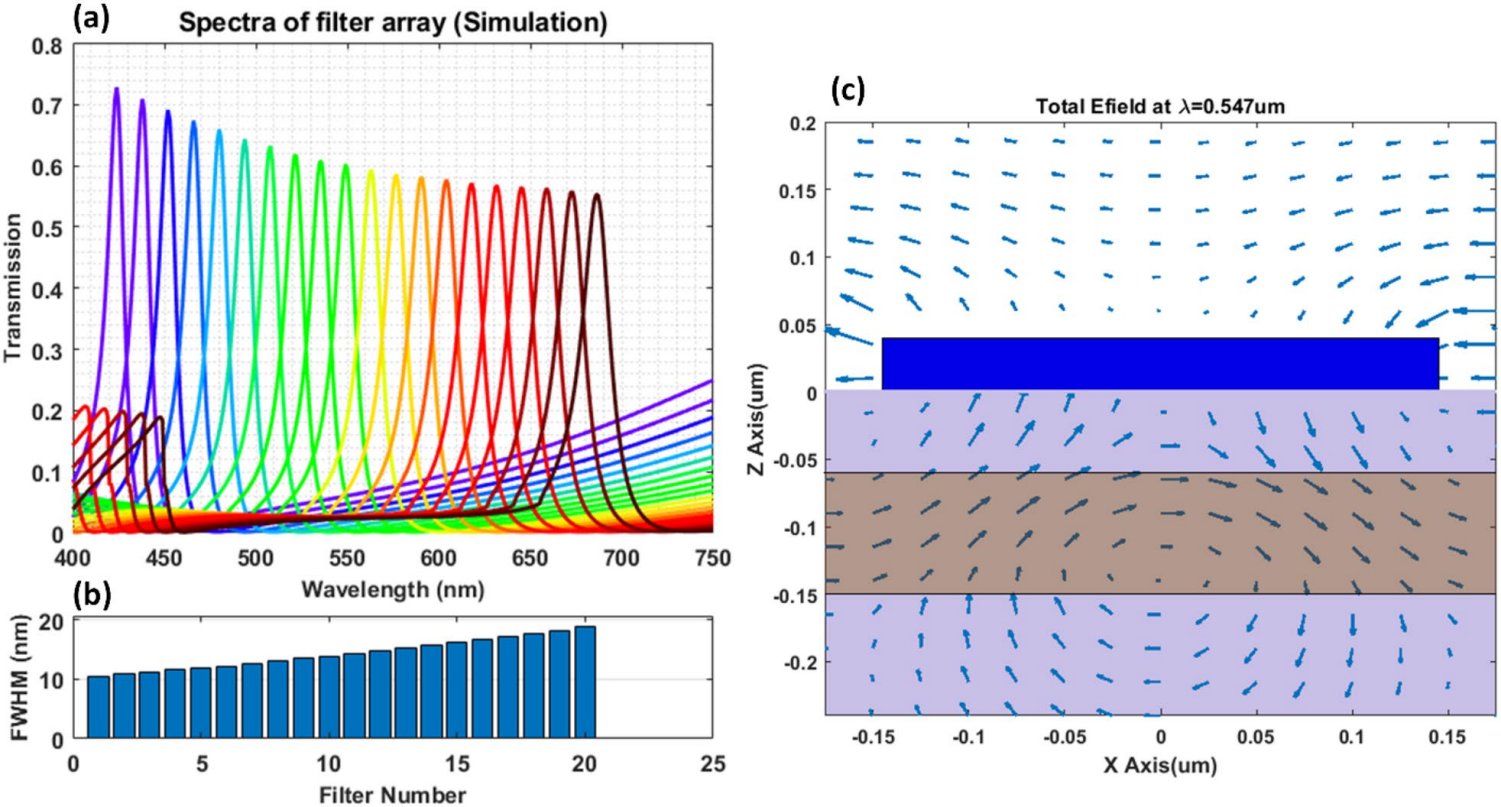
(**a**) Simulated spectra of filter array (20 filters) with period varied from 260 to 450 nm at 10 nm steps. (**b**) FWHM of each filter. (**c**) Total E-field plot of a unit cell of filter No. 10 at peak wavelength of 547 nm.

An array of 20 filters (in 4 × 5 matrix) were designed using RCWA and filter spectra was optimized for 10–20 nm bandwidth The distance between the grating and slab waveguide was optimized to achieve such bandwidth. Figure 2a shows the simulated spectra of all 20 filters and Fig. 2b shows the full wave half maximum (FWHM) distribution of the 20 filters. In addition, Fig. 2c shows total E-field quiver plot of one of the filters at the peak wavelength at 547 nm.

### Filter array fabrication and characterization

**Figure 3:**
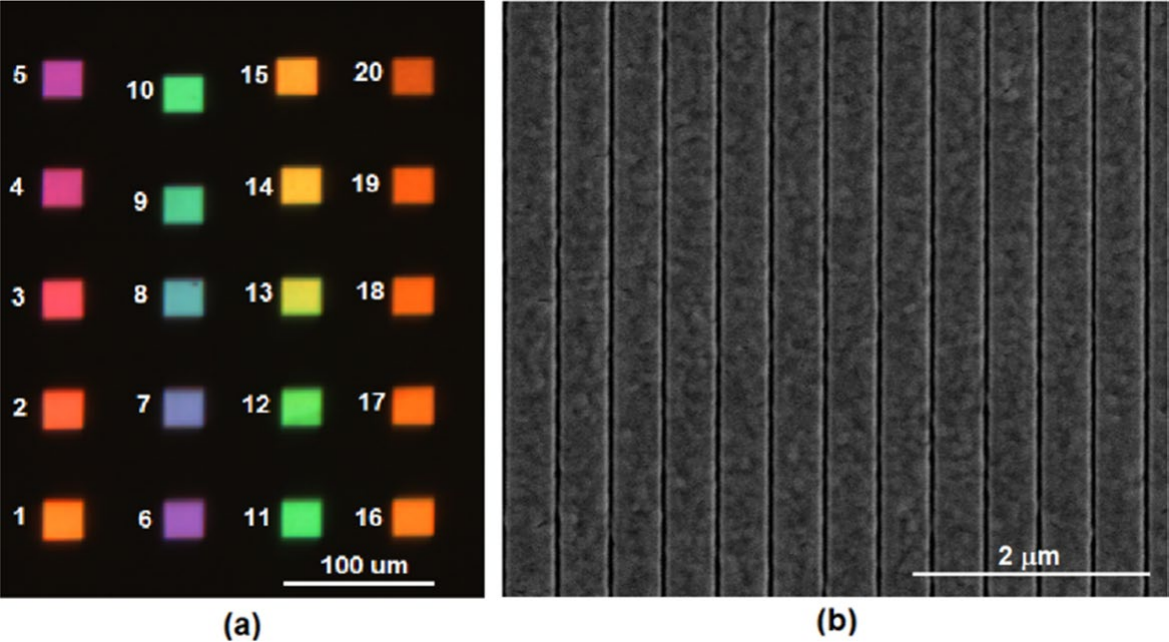
(**a**) RGB image of filter array with annotated filter numbers and (**b**) SEM image of a section of one of the filters.

The array was fabricated as described in methods section using Plasma Enhanced Chemical Vapor Deposition (PECVD), Evaporation and Focused Ion Beam (FIB) technique. Figure 3 below shows RGB image of the filter array taken using optical microscope and SEM image of a section of one of the filters.

**Figure 4:**
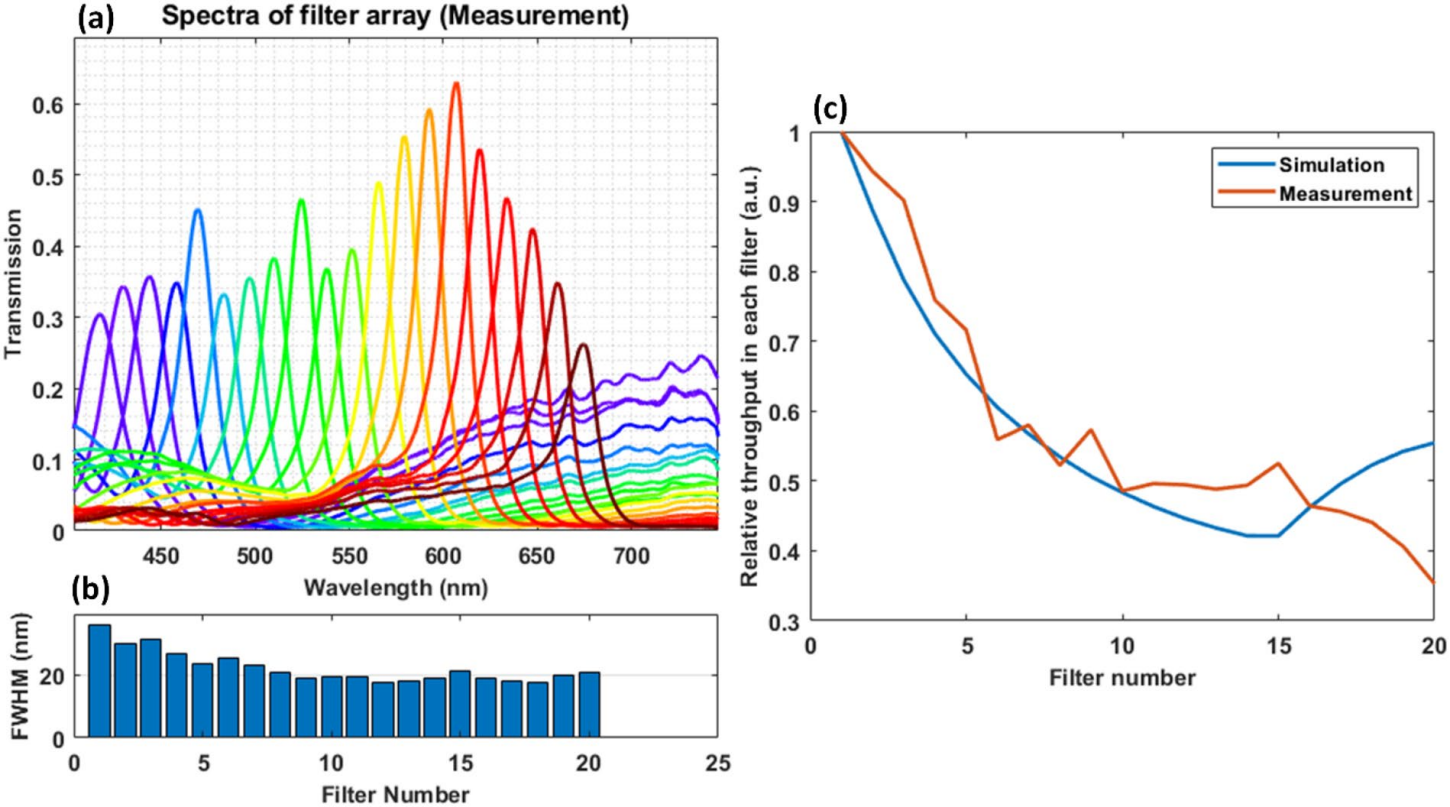
(**a**) Measured filter array spectra, (**b**) FWHM of each filter, and (**c**) relative throughput in each filter comparison between simulated spectra and measured spectra.

The filters were then characterized using test setup shown in Fig. 6 in methods section, after replacing the camera with spectrometer probe. Figure 4a below shows the characterized filter array spectra as measured by the spectrometer and Fig. 4b shows corresponding FWHM of each filter. The measured filter spectra are very similar to simulated results, except for FWHM at shorter wavelengths. The broadening of the transmission peak can be attributed to more loss at shorter wavelengths compared to material properties used in the simulation environment. Figure 4c shows relative throughput (area under the curve) through each filter compared between simulation and measurement. As can be seen filter throughput between simulation and measurement is consistent, which indicates that the filters at shorter wavelengths are broadened and hence look attenuated but the total area under each curve shows consistent trend.

### Spectroscopy and spectral reconstruction

**Figure 5:**
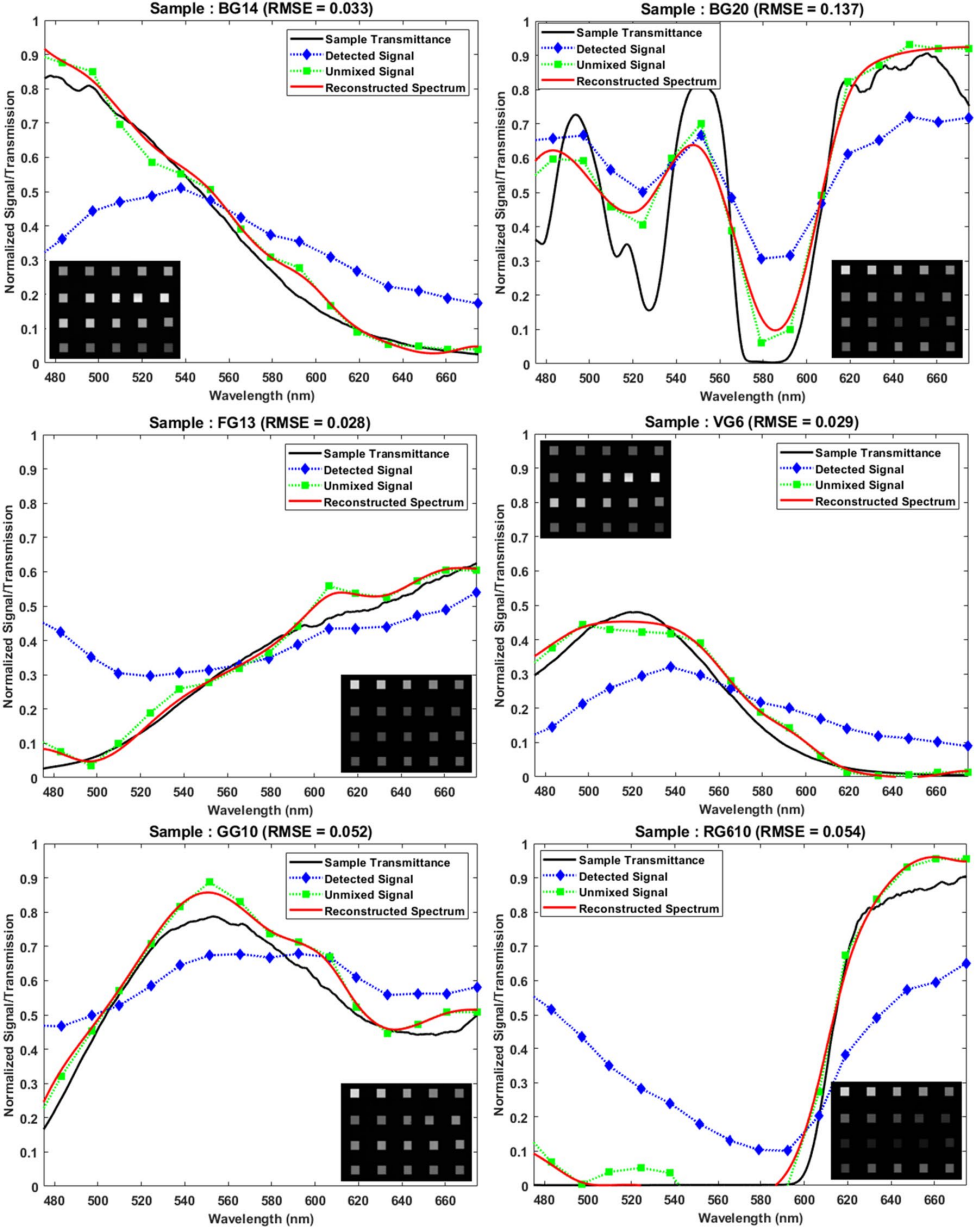
Results of spectral reconstruction of 6 samples using snapshot images (shown in insets) through the color filter array. Black curves show sample transmission (ground truth), blue curves show the signal extracted from images, green curves show the signals after unmixing algorithm and red curves show the reconstructed spectra.

A set of 6 colored glass filters were used as samples to characterize the performance of the designed color filter array and reconstruction algorithm. The details of fabrication, testing and algorithm is described in methods section. Figure 5 below shows the results of reconstruction of transmission spectra of these samples based on the snapshot images taken with a monochromatic camera.

The second sample (BG20) was purposely chosen to test the limits of the system, since it has many sharp transitions and narrow features. It is noted that except for sample 2, the RMSE is less than 0.054 for rest of the samples. The performance degradation in sample two is expected and shows the limiting performance of the spectroscopy system.

## Discussion

The use of the color filter array to detect power in each spectral band and reconstruction of original spectra poses several challenges. Firstly, the spectrum of each filter has prolonged tail at the longer wavelengths and Rayleigh anomaly peaks at the short wavelengths, which causes spectral mixing. For example, in Fig. 2a the first filter can transmit light in the range of 425 nm to 450 nm as well as that beyond 600 nm. Hence any reading on this color channel could be due to either of these spectral bands. Due to such mixing, as can be seen in Fig. 5, the detected signal is quite different from the original sample spectra. Hence an unmixing algorithm was developed to band-wise unmix detected signals. Secondly, the reconstruction of original spectra (at 1 nm steps) from under-sampled data (only 20 filters in entire visible range) can be framed in the linear algebraic terms as solving under-determined least square problem, which has infinite many solutions. Hence a unique regularization method using difference operator was used for reconstructing smooth spectra from unmixed signals.

Although sample spectra could be recovered there are several sources of error that limits the performance, some of which are:

1. There is inherent assumption in proposed algorithm that spectrum is constant within each band. This introduces some error.
2. Some of the spectra, especially sample two BG20, contains very sharp features which has frequency components higher than that Nyquist frequency and hence some of the information is aliased, which degrades reconstruction accuracy in this filter.
3. The reconstruction is based on filter characterization and measurement errors in filter characterization can result in reconstruction error. The filters were characterized by coupling imaged filter patterns onto an optical probe of the spectrometer and hence variations in coupling efficiency could results in errors in filter characterization, which leads to errors in reconstruction.
4. Lastly, there is inherent cross-talk between filters due to proximity and due to presence of contiguous slab waveguide. The slab waveguide provides means for some of the rejected power from one filter to appear in the other filters. However, this effect is partly captured during filter characterization and as long as filters are characterized in-situ including such cross-talk, it doesn’t affect the reconstruction. However due to cross-talk, the characterized filter array spectra are expected to be different from simulation.

## Methods

The filter array was designed in DiffractMod software from Synopsys which uses Rigorous Coupled Wave Analysis (RCWA) technique. The periods of the filters were varied from 260 to 450 nm at 10 nm steps. The filters were designed to be 25 µm × 25 µm in size separated by 50 µm spacing forming 4 × 5 mosaic pattern. The design was fabricated on 500 µm thick Quartz substrate on which 90 nm of Silicon Nitride was deposited using Plasma Enhanced Chemical Vapor Deposition (PECVD) followed by 60 nm of Silicon Dioxide using same PECVD tool. Then 40 nm of Aluminum was deposited using evaporation. The filter array was then patterned using Focused Ion Beam (FIB) milling using Gallium ions with 30 keV energy. The writing was performed at 30 pA write current and with scan speed such that dose is 35 mC/cm^2^.

The samples (6 colored glass filter from Newportglass^35^) were measured by passing white light through the samples and the Plasmonic Filter Array and capturing snapshot images of the Filter Array using an objective lens. The Amscope HL250-AY was used as white light source. The gooseneck of the lamp was held on a mounting fixture and the light was used to illuminate a variable aperture. The light from the aperture was then collected using an aspherical achromatic collimating lens (APAC15) with effective focal length of 30 mm from Newport Optics. The light was then passed through visible light filter (FESH750) with cutoff at 750 nm. Then a wire grid polarizer (WP25M-VIS) was used to polarize the illumination. Then the samples were placed in the light path with a mounting fixture. Then the glass slide with plasmonic filter array was mounted on X–Y stage and placed in the optical axis. Then a MPlan 10 × objective was used to image the filter array from the other side onto DMK21AU04 monochromatic camera. The background illumination was reduced by conducting the experiment in a dark room and by properly shielding stay light. The camera was connected to a computer in which automatic exposure time adjustment algorithm was run to keep the signals in images around 50% of the camera’s dynamic range.

**Figure 6:**
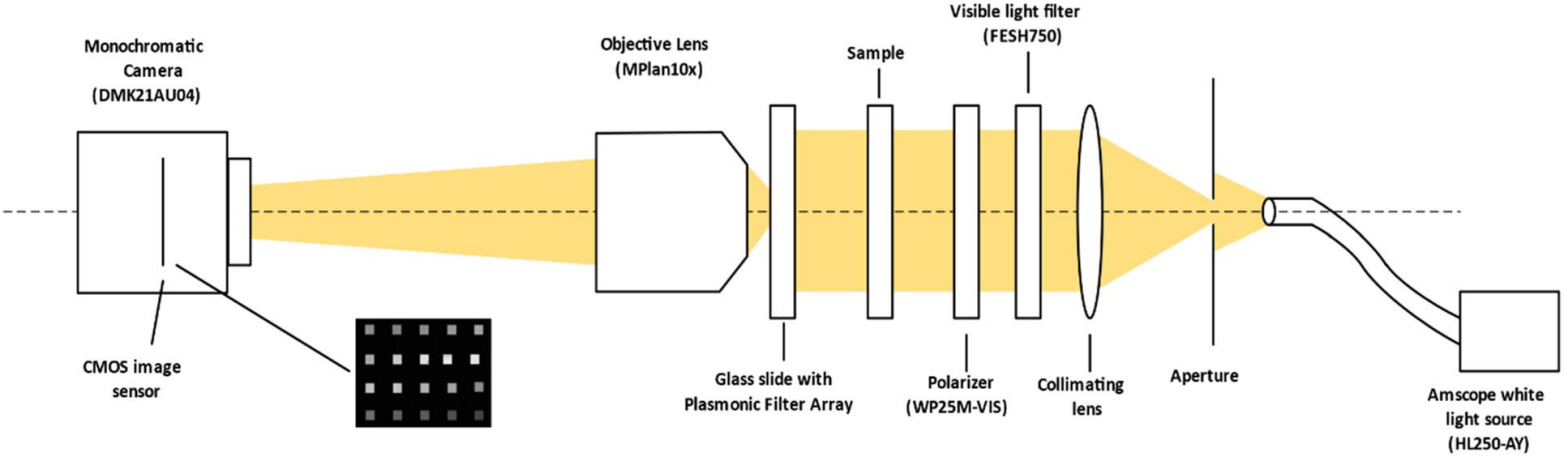
Test setup for taking snapshot images of samples.

Figure 6 below shows the test setup used for sample measurement.

After sample images were taken, the filter array was characterized by replacing the camera with Ocean Optics USB2000+ spectrometer and by removing the samples. The images of each filter were directly coupled into the open end of the optical probe of the spectrometer by mounting the probe on a X/Y translation stage. The open end of the probe was placed at the image plane.

The images were then postprocessed for background subtraction and segmentation. Then the detected signals were calculated based on average of the pixel values within region of interest of each filter in each image. These values represent blue curves with diamond markers in Fig. 5. Then the Algorithm 1 (described below) was used to unmix various spectral regions.

For unmixing algorithm, we consider the signal acquisition in two steps. Firstly, the illumination passes through an idealized set of filters (without much overlaps) and then secondly these signals are mixed at various proportions to produce detected signals. Such operation can be represented by:

1 $$ {\text{D}}_{{\text{N}}} = {\text{M}}_{{{\text{N}} \times {\text{N}}}} \times {\text{G}}_{{{\text{N}} \times n}} \times \left( {S_{n} \bullet I_{n} } \right) $$

Here ${\text{S}}_{{\text{n}}}$ is sample transmittance, $I_{n}$ is illumination, ${\text{G}}_{{{\text{N}} \times n}}$ is idealized filter transmission matrix, $M_{N \times N}$ is the mixing matrix and $D_{N}$ is the detected signal. Here × signifies matrix multiplication and • signifies element-wise product. The mixing matrix $M_{M \times N}$ is then computed using following algorithm.
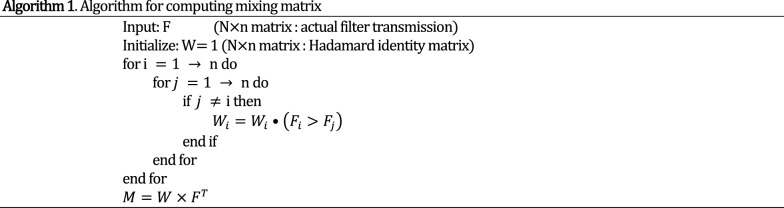


Then ${\text{M}}$ is a N × N matrix representing mixing proportions from each filter to every other filter. This is an invertible square matrix. This assumes that spectrum is constant across each band, which for a set of narrow filters is a good approximation. Also, this implies the product ${\text{M}}^{ - 1} \times {\text{F}}$ results in idealized filter transmission matrix. Now unmixing can be performed using following operation.

2 $$ {\text{D}}_{{\text{U}}} = {\text{M}}_{{{\text{N}} \times {\text{N}}}}^{ + } \times {\text{D}}_{{\text{N}}} $$

where ${\text{D}}_{{\text{U}}}$ is unmixed detected signals per color channel and + operation is regularized pseudo-inverse. A regularized pseudo-inverse is used instead of matrix inversion to find a smooth low frequency signal and to discard oscillatory and unrealistic solutions.

Now once the signals are unmixed, the reconstruction can be performed by solving least square problem using similarly regularized pseudo-inverse. Firstly ${\text{G}}_{{{\text{N}} \times n}}$ is computed as factor of the characterized filter transmission matrix ${\text{F}}_{{{\text{N}} \times n}}$ using following equation.

3 $$ {\text{G}}_{{{\text{N}} \times n}} = {\text{M}}_{{{\text{N}} \times {\text{N}}}}^{ + } \times {\text{F}}_{{{\text{N}} \times n}} $$

Now such idealized filter spectra can be used to reconstruct spectra from the unmixed signals as below.

4 $$ {\text{R}}_{{\text{n}}} = \frac{{{\text{G}}_{{{\text{N}} \times {\text{n}}}}^{ + } \times {\text{D}}_{{\text{U}}} }}{{{\text{R}}_{{{\text{n}}1}} }} $$

Here again the inverse is regularized pseudo-inverse and $R_{n1} ( = {\text{G}}_{{{\text{N}} \times n}} \times D_{U1}$) is the first reconstructed spectrum without any samples. The regularized pseudo-inverse is computed using following equation:

5 $$ A^{ + } = \left( {A^{T} \times {\text{A}} - {\text{kG}}} \right) \times A^{T} $$

where k is the regularization parameter and G is second order difference operator given by:

6 $$ G = \left[ {\begin{array}{*{20}l} {\begin{array}{*{20}c} 1 & { - 2} & 1 \\ \end{array} } \hfill & \cdots \hfill & {\begin{array}{*{20}c} 0 & 0 & 0 \\ \end{array} } \hfill \\ \vdots \hfill & \ddots \hfill & \vdots \hfill \\ {\begin{array}{*{20}c} 0 & 0 & 0 \\ \end{array} } \hfill & \cdots \hfill & {\begin{array}{*{20}c} 1 & { - 2} & 1 \\ \end{array} } \hfill \\ \end{array} } \right] $$

The regularization parameter k determines the strength of smoothing by penalizing highly oscillatory solutions. Choosing a k value equal to zero leads to the case of non-regularized pseudo-inverse or least square error minimum norm solution, while choosing higher k value leads to smoother solution. The value of k was optimized with many iterations to seek for optimum solution. In inversion operation in Eq. (2), the k value was 100 and that in Eq. (4) it was 1.

For computing transmission spectra, the reconstructed signal hence calculated, includes a signal without any samples, which is considered as illumination signal and when divided by such signal, the sample transmittance ${\text{S}}_{{\text{n}}}$ can be recovered as ${\text{R}}_{{\text{n}}}$. In Fig. 5, black curves are ${\text{S}}_{{\text{n}}}$, curves with blue markers are ${\text{D}}_{{\text{N}}}$, curves with green markers are ${\text{D}}_{{\text{U}}}$ and red curves are ${\text{R}}_{{\text{n}}}$. The reconstructed spectra are very similar to the original sample spectra and root mean squared error is less than 0.137 across all samples.

## Data Availability

The data collected from testing in this research study are available from the corresponding author on reasonable request.

## References

[1] Panasyuk SV (2007). Medical hyperspectral imaging to facilitate residual tumor identification during surgery. Cancer Biol. Ther..

[2] Lu, G. & Fei, B. Medical hyperspectral imaging: A review. *J. Biomed Opt.* **19**, 010901 (2014). 10.1117/1.JBO.19.1.010901 PMC3895860 24441941

[3] Alfano RR, Das BB, Cleary J, Prudente R, Celmer E (1991). Light sheds light on cancer–distinguishing malignant tumors from benign tissues and tumors. Bull. N. Y. Acad. Med..

[4] Clark RN, Roush TL (1984). Reflectance spectroscopy: Quantitative analysis techniques for remote sensing applications. J. Geophys. Res. Solid Earth.

[5] Govender M, Chetty K, Bulcock H (2007). A review of hyperspectral remote sensing and its application in vegetation and water resource studies. Water Sa.

[6] Adam E, Mutanga O, Rugege D (2010). Multispectral and hyperspectral remote sensing for identification and mapping of wetland vegetation: A review. Wetl. Ecol. Manag..

[7] Deaton, B. C. & Balsam, W. L. Visible spectroscopy—A rapid method for determining hematite and goethite concentration in geological materials. *J. Sediment. Res.* **61** (1991).

[8] Gowen AA, O’Donnell CP, Cullen PJ, Downey G, Frias JM (2007). Hyperspectral imaging—An emerging process analytical tool for food quality and safety control. Trends Food Sci. Technol..

[9] Delaney JK (2010). Visible and infrared imaging spectroscopy of picasso’s harlequin musician: Mapping and identification of artist materials in situ. Appl. Spectrosc..

[10] Balas C (2003). A novel hyper-spectral imaging apparatus for the non-destructive analysis of objects of artistic and historic value. J. Cult. Herit..

[11] Moghareh Abed, F. Pigment identification of paintings based on kubelka-munk theory and spectral images. *Ph. D. Thesis* (2014)

[12] Fischer C, Kakoulli I (2006). Multispectral and hyperspectral imaging technologies in conservation: current research and potential applications. Stud. Conserv..

[13] Hirakawa, K. & Wolfe, P. J. Spatio-spectral sampling and color filter array design. In *Single-Sensor Imaging*, 157–172 (CRC Press, 2018).

[14] Maloney LT, Wandell BA (1986). Color constancy: A method for recovering surface spectral reflectance. JOSA A.

[15] Connah, D., Hardeberg, J. Y. & Westland, S. Comparison of linear spectral reconstruction methods for multispectral imaging. In *2004 International Conference on Image Processing, 2004. ICIP’04.*, vol. 3, 1497–1500 (IEEE, 2004).

[16] Hardeberg, J. Y. Recent advances in acquisition and reproduction of multispectral images. In *2006 14th European Signal Processing Conference*, 1–5 (IEEE, 2006).

[17] Parmar, M., Lansel, S. & Wandell, B. A. Spatio-spectral reconstruction of the multispectral datacube using sparse recovery. In *2008 15th IEEE International Conference on Image Processing*, 473–476 (IEEE, 2008).

[18] Shen H-L, Cai P-Q, Shao S-J, Xin JH (2007). Reflectance reconstruction for multispectral imaging by adaptive wiener estimation. Opt. Express.

[19] Heikkinen V (2008). Evaluation and unification of some methods for estimating reflectance spectra from rgb images. JOSA A.

[20] Cheung V, Westland S, Li C, Hardeberg J, Connah D (2005). Characterization of trichromatic color cameras by using a new multispectral imaging technique. JOSA A.

[21] Shen H-L, Xin JH, Shao S-J (2007). Improved reflectance reconstruction for multispectral imaging by combining different techniques. Opt. Express.

[22] Ximea hyperspectral cameras based on usb3, https://www.ximea.com/en/products/xilab-application-specific-oem-custom/hyperspectral-cameras-based-on-usb3-xispec. (2021).

[23] Lambrechts, A. *et al.* A cmos-compatible, integrated approach to hyper-and multispectral imaging. In *2014 IEEE International Electron Devices Meeting*, 10–5 (IEEE, 2014).

[24] Bayer, B. E. Color imaging array. *United States Pat. 3,971,065* (1976).

[25] Yokogawa S, Burgos SP, Atwater HA (2012). Plasmonic color filters for cmos image sensor applications. Nano Lett..

[26] Bravo-Abad J (2006). How light emerges from an illuminated array of subwavelength holes. Nat. Phys..

[27] Burgos SP, Yokogawa S, Atwater HA (2013). Color imaging via nearest neighbor hole coupling in plasmonic color filters integrated onto a complementary metal-oxide semiconductor image sensor. ACS Nano.

[28] Qiang, Z. *et al.* Fano filters based on transferred silicon nanomembranes on plastic substrates. *Appl. Phys. Lett.* **93**, 061106 (2008).

[29] Shrestha VR, Lee S-S, Kim E-S, Choi D-Y (2014). Aluminum plasmonics based highly transmissive polarization- independent subtractive color filters exploiting a nanopatch array. Nano Lett..

[30] Xu T, Wu Y-K, Luo X, Guo LJ (2010). Plasmonic nanoresonators for high-resolution colour filtering and spectral imaging. Nat. Commun..

[31] Li E, Chong X, Ren F, Wang AX (2016). Broadband on-chip near-infrared spectroscopy based on a plasmonic grating filter array. Opt. Lett..

[32] Duempelmann L, Gallinet B, Novotny L (2017). Multispectral imaging with tunable plasmonic filters. ACS Photon..

[33] Kaplan AF, Xu T, Jay Guo L (2011). High efficiency resonance-based spectrum filters with tunable transmission bandwidth fabricated using nanoimprint lithography. Appl. Phys. Lett..

[34] Lee K-T, Seo S, Guo LJ (2015). High-color-purity subtractive color filters with a wide viewing angle based on plasmonic perfect absorbers. Adv. Opt. Mater..

[35] Newport industrial glass. http://www.newportglass.com/schott.htm. (2021).

